# Race, explicit racial attitudes, implicit racial attitudes, and COVID-19 cases and deaths: An analysis of counties in the United States

**DOI:** 10.1371/journal.pone.0242044

**Published:** 2020-11-18

**Authors:** George B. Cunningham, Lisa T. Wigfall

**Affiliations:** 1 Department of Health and Kinesiology, Center for Sport Management Research and Education, Texas A&M University, College Station, TX, United States of America; 2 Department of Health and Kinesiology, Transdisciplinary Center for Health Equity Research, Texas A&M University, College Station, TX, United States of America; University of Connecticut, UNITED STATES

## Abstract

**Objectives:**

To examine the potential moderating effects of explicit racial attitudes and implicit racial attitudes on the relationship between percent of Black county residents and COVID-19 cases and deaths.

**Methods:**

We collected data from a variety of publicly available sources for 817 counties in the US. (26% of all counties). Cumulative COVID-19 deaths and cases from January 22 to August 31, 2020 were the dependent variables; explicit racial attitudes and implicit racial attitudes served as the moderators; subjective poor or fair health, food insecurity, percent uninsured, percent unemployed, median family income, percent women, percent of Asian county resident, percent of Hispanic county residents, and percent of people 65 or older were controls.

**Results:**

The percent of Black county residents was positively associated with COVID-19 cases and deaths at the county level. The relationship between percent of Black residents and COVID-19 cases was moderated by explicit racial attitudes and implicit racial attitudes.

**Conclusions:**

Implicit racial attitudes can take on a shared property at the community level and effectively explain racial disparities. COVID-19 cases are highest when both the percent of Black county residents and implicit racial attitudes are high.

## Introduction

Whereas everyone has an exposure risk for COVID-19, the severity of illness varies.

There is a growing body of evidence that suggests racial and ethnic minorities are more affected by severe illness from COVID-19 compared to White people [1–4]. Even more alarming is the fact that some researchers have shown that Black individuals are at a higher risk than their White counterparts of having severe illness from COVID-19 that requires hospitalization [3,5]. In the United States (US), this is not surprising because Blacks/African American people were already disproportionately affected by underlying medical conditions such asthma, cardiovascular disease (CVD), chronic kidney disease, chronic obstructive pulmonary disease (COPD), diabetes, hypertension or high blood pressure (HBP), human immunodeficiency virus (HIV), obesity, and sickle cell disease before COVID-19 [6]. What is less clear to date, however, are the factors that may be associated with these racial disparities in adverse health outcomes from COVID-19 [7].

The purpose of this research was to extend the existing scholarship related to race and COVID-19 by empirically considering the role of explicit and implicit racial attitudes. In this study, we draw from publicly available data to examine the role of racial attitudes on COVID-19 cases and deaths, at the county level. Our results indicate that as the percent of Black county residents increases, so too do COVID-19 cases and deaths. We hypothesize that explicit and implicit racial attitudes uniquely explain COVID-19 cases and deaths, and serve to moderate the relationship between the percent of Black county residents and COVID-19 outcomes.

### Theoretical framework

In the current study, we consider the influence of racial attitudes and how they might affect the relationship between the percent of Black county residents and COVID-19 cases and deaths. Moderators influence the relationship between predictor and outcome variables and, in doing so, offer unique information about when and under what conditions phenomena might occur [8]. Consistent with current social psychological research and public health scholarship [9,10], we consider the influences of both explicit and implicit attitudes toward racial minorities.

Explicit racial attitudes are those of which people are aware and that they consciously, deliberately maintain [11]. This form of bias is consistent with the notion that prejudice represents negative attitudes held toward a group of people or those presumed to be in that group (e.g., [12]). Explicit racial bias might take the form of racial slurs or holding negative attitudes toward racial minorities. In much of the US, publicly espousing explicit racial prejudice is contrary to social norms [13]; nevertheless, people still do harbor such attitudes and, in some cases, openly express them.

Implicit racial attitudes take a different form than do explicit attitudes. They reflect automatic responses that manifest when an external stimulus corresponds with an individual’s association set that link the stimulus and particular characteristics [14]. Though implicit attitudes represent automatic associations, people are aware of their own implicit biases and are able to predict their occurrence with some accuracy [15]. Several scholars have argued that implicit racial attitudes are more prevalent in society than are explicit attitudes [16] and might be a better predictor of less controllable behaviors [17], especially among those who are not motivated to avoid bias.

#### Racial attitudes at the at the community level

To date, most of the research focusing on explicit and implicit racial attitudes has occurred at the individual level of analysis; however, the relationship with individual-level behaviors is tenuous [18]. On the other hand, Payne and colleagues [19,20] have shown that prejudice can take on a shared property at the community level. Members of a community—whether a city, metropolitan area, county, or even state—generally have similar social interactions, exposures to media, and collective experiences, all of which can shape their explicit and implicit attitudes. The collective experiences can influence how any one individual reacts to a particular situation, and therefore, contextual factors likely attenuate the relationship between bias and outcomes at the individual levels. However, when taken as a whole, aggregate responses should converge such that people within a given community respond in similar ways. In this way, prejudice represents a bias of the crowd [19,20].

Past researchers focusing on racial bias and health outcomes have predominantly done so at the individual level of analysis (for examples, see [21,22]). However, for many of the reasons articulated by Payne et al. [19,20]., bias captured at the community-level is likely to offer better explanatory power (see also [23]). Consistent with this position, community-level racial attitudes are linked with racial differences in infant health outcomes [24], Black individuals’ death rates due to cardiovascular disease [25], and mortality risk [26], as well as other important community outcomes, such as lethal force in policing [27], punishment of Black school children [28], and reactions to social justice movements [29], among others.

#### Hypotheses

The collective evidence suggests that race is likely to influence COVID-19 risk, such that as the percent of racial minorities increases, so too do COVID-19-related cases and deaths [1–4]. Researchers have also shown that the unique effects of race and ethnicity remain even after taking into account individual and neighborhood factors [5], leading to calls for further research aimed at identifying underlying predictive mechanisms. Community-level racial attitudes might offer such predictive value, as they are related to a variety of negative health outcomes for Black residents [24–26].

Building from this evidence, we first predicted that explicit racial attitudes would predict COVID-19 cases (Hypothesis 1) and deaths (Hypothesis 2), such that as explicit racial attitudes increased, so too would the COVID-19 related outcomes. We hypothesized similar effects for implicit racial attitudes, such that we expected they would hold a significant, positive association with COVID-19 cases (Hypothesis 3) and deaths (Hypothesis 4). Finally, it is possible that explicit racial and implicit attitudes moderate the relationship between the percent of Black county residents and subsequent health outcomes. In this case, the aforementioned relationship might be stronger when explicit and implicit attitudes are high, relative to when they are low. This reasoning is consistent with other scholarship showing that racial attitudes and biases can interact with community demographics to predict health outcomes [30]. Given these possibilities, we hypothesized that explicit racial attitudes would moderate the relationship between the percent of Black county residents and COVID-19 cases (Hypothesis 5) and deaths (Hypothesis 6), such that the associations would be stronger when explicit racial attitudes are high. Turning to the next hypotheses, we predicted that implicit racial attitudes would moderate the relationship between the percent of Black county residents and COVID-19 cases (Hypothesis 7) and deaths (Hypothesis 8), such that the relationships will be stronger when implicit racial bias is high.

## Method

### Data sources and variables

We collected data at the county level in the United States. Of the 3143 county-equivalents (e.g., counties, parishes, boroughs) in the US, we had complete data for 817 (26% of all counties), with every state and the District of Columbia represented. As outlined in the following sections, we drew from a variety of publicly available sources.

#### COVID-19 cases and deaths

We collected COVID-19 cases and deaths for each county for the USAFacts website (https://usafacts.org/visualizations/coronavirus-covid-19-spread-map/). This site uses a county-level tracker to collect data for each day. For the current analysis, we had cumulative cases and deaths observations from January 22, 2020 through August 31, 2020 (222 days). We converted each count to reflect the total the number of cases (*Cases*) or deaths (*Deaths*) per 100,000 county residents.

#### Black county residents

We drew from the Robert Wood Johnson-supported County Health Rankings & Roadmaps website (www.countyhealthrankings.org) to determine the percent of Black county residents (*Percent Black*). This site draws from US Census Bureau estimates to provide the demographic characteristics for each county in the US, including the percentage of Black residents.

#### Racial attitudes

We included two measures for racial attitudes: *Explicit Racial Attitudes* and *Implicit Racial Attitudes*, both of which were collected from Project Implicit. Harvard University researchers have, for years, collected bias and prejudice data from people around the world through the Project Implicit project (http://implicit.harvard.edu). After anonymizing the data, they then make it publicly available on the Center for Open Science website (https://osf.io/vp5m2/). It is from this website that we collected data for the 2019 year. The dataset includes information about the state and county of the participants.

We assessed *Explicit Racial Attitudes* using the Feelings Thermometer. Participants responded to two items: “please rate how warm or cold you feel towards White people” and “please rate how warm or cold you feel towards Black people.” Both items were anchored by a scale including 0 = coldest feelings, 5 = neutral, 10 = warmest feelings. Consistent with Hehman et al. [27], Leitner et al. [25], O’Shea, Watson, Brown, and Fincher [31], among others, we subtracted ratings toward Black people from the ratings toward White people to create a single measure of *Explicit Racial Attitudes*.

We examined *Implicit Racial Attitudes* through the Implicit Association test developed by Greenwald, McGhee, and Schwartz [32]. This measure “assesses strengths of associations between concepts by observing response latencies in computer-administered categorization tasks” ([33], p. 18). Researchers calculate participant responses times between various associations to determine the relative preference for or against Black people or White people. Thus, the focus is on automatic associations instead of an explicit response. Greenwald et al. [33] offered evidence for the test-retest reliability, internal consistency, and various forms of validity evidence.

#### Controls

We included a number of control variables that might influence the residents’ health or that are related to COVID-19 prevalence. Data were gathered from the County Health Rankings & Roadmaps website, and we also list the data source from which they drew the data and formed their estimates. First, a number of pre-existing health conditions that are linked with greater COVID-19 risks and mortality [34]; thus, we controlled for the percent of county participants whose subjective ratings were *Poor or Fair Health*. Others have also used their measure to assess community-level health [35,36]. Next, food a lack of healthy food options is related to poor health outcomes [37] and a potential contributor to COVID-19 prevalence [38]. Thus, we controlled for the percent of county residents experiencing *Food Insecurity* using the data from the Map the Meal Gap. Third, a lack of insurance can negatively affect preventative health and disease treatment [39]. People who lack insurance might also face more negative COVID-19 outcomes [40]. Thus, we accounted for the percent of the county who are *Uninsured*, with data coming from the Small Area Health Insurance Estimates. Given the influence of economic stability on health [41] and COVID-19 risk [42], we included the percent of residents who were *Unemployed*, with data from the Bureau of Labor Statistics. Further, income is predictive of COVID-19 outcomes, as people in poor communities are countries bear a larger burden than do their wealthier counterparts [43]. As such, we controlled for the log of the median family income among county residents. Finally, race, gender, and age are reliably associated with COVID risks [44,45], so we controlled for the percent of county residents who were *Percent Women*, *Percent Asian*, *Percent Hispanic*, and *Percent 65 or Older*.

### Empirical analysis

Previous researchers have aggregated data at various cutoff points (e.g., *n* = 20 for [24]; *n* = 100 for [46]; and *n* = 150 for [27]). In determining the most appropriate cutoff point, we ran preliminary analyses for a minimum of 20, 50, 100, and 150 respondents per county. To examine precision, we computed the coefficient of variation for the Explicit Racial Attitudes and Implicit Racial Attitudes at each cutoff point and then compared the percent increase in precision. The coefficient of variation scores were substantially smaller in counties with at least 50 respondents, relative to those with 20. The increased precision was only marginally better when to 100 and then to150. We then compared representativeness of the sample. When 20 respondents served as the cutoff point, multiple counties in each state were represented in the dataset. This was also the case when moving to a minimum of 50 respondent per county. When we moved to 100 cases, there were no counties from one state, and only a single county from other states. This also occurred when using 150 cases as the cutoff. Given these patterns, we chose 50 as the minimum number of respondents per county. As a result, the data were reduced from 875,209 individual responses to the aggregate responses representing 817 US counties.

Next, there was considerable variability in the number of Project Implicit respondents per county (50 to 13,003). To account for this unevenness in the responses per county, we followed Nosek et al. [46] by constructing the log of the inverse weights based on standard errors. Consistent with Nosek et al. [46], we averaged the weights for the three racial attitudes measures to create a single weighting variable. This approach allows for more reliable estimates in the counties with more respondents.

We computed bivariate correlations for all variables. We standardized the predictor and moderating variables (consistent with [46]) and tested the hypotheses through moderated regression analysis, following Cohen et al.’s [47] guidelines. Where significant interactions existed, we computed simple slopes and plotted the nature of the interactions. *Cases* and *Deaths* served as the dependent variables, respectively, in the analyses. Preliminary analyses indicated that when all controls, first-order effects, and the interaction terms were entered into the regression analysis simultaneously, the variance inflation factor was 18.45. This is greater than the 10.00 cutoff point recommended by Hair, Black, Babin, Anderson, and Tatham [48], thereby signaling multicollinearity was a concern. Thus, we ran separate analyses for *Explicit Racial Attitudes* and *Implicit Racial Attitudes*. This approach is also consistent with Riddle and Sinclair [28].

## Results

### Descriptive statistics

The means, standard deviations, and bivariate correlations are available in Table 1. Overall, the counties included in the sample averaged 1466.37 (*SD* = 941.35) total COVID-19 *Cases* and 38.12 (*SD* = 42.20) total *Deaths* through the end of August 2020. Both COVID-19 variables held statistically significant associations with most of the control and predictor variables. Of note was the positive bivariate associations between *Percent Black* and both *Cases* (*r* = .47) and *Deaths* (*r* = .32). These associations were higher than the corresponding relationships observed for *Percent Asian* or *Percent Hispanic*. Finally, *Explicit* and *Implicit Racial Attitudes* held a significant, positive association (*r* = .72).

**Table 1:** 

Bivariate correlations, means, and standard deviations							
Variable	1	2	3	4	5	6	7
1 Fair or Poor Health	--						
2 Uninsured	.56***	--					
3 Unemployed	.47***	.07*	--				
4 Food Insecure	.61***	.40***	.32***	--			
5 Log Household Income	-.67***	-.33***	-.38***	-.71***	--		
6 Percent 65 or Older	-.15***	-.10**	.08*	-.12**	-.17***	--	
7 Percent Female	.12***	.10**	-.02	.14***	-.04	.19***	--
8 Percent Asian	-.17***	-.12**	-.11**	-.13***	.45***	-.27***	.03
9 Percent Hispanic	.42***	.44***	.36***	-.03	.00	-.26***	-.08
10 Percent Black	.40***	.31***	.13***	.54***	-.20***	-.24***	.43***
11 Explicit Racial Attitudes	-.39***	-.36***	-.22***	-.40***	.09	.30***	-.25***
12 Implicit Racial Bias	-.30***	-.31***	-.14***	-.43***	.12**	.26***	-.14***
13 Cases	.51***	.50***	.21***	.24***	-.12***	-.29***	.08
14 Deaths	.23***	.09*	.22***	-.01	.09	-.05	.27***
*Mean*	15.98	9.16	3.85	12.18	11.03	16.65	50.60
*SD*	3.50	4.16	1.22	3.34	0.24	3.98	1.20
Unweighted Bivariate Correlations, Means, and Standard Deviations							
Variable	8	9	10	11	12	13	14
1 Fair or Poor Health							
2 Uninsured							
3 Unemployed							
4 Food Insecure							
5 Log Household Income							
6 Percent 65 or Older							
7 Percent Female							
8 Percent Asian	--						
9 Percent Hispanic	.23***	--					
10 Percent Black	.05	-.06	--				
11 Explicit Racial Attitudes	-.28***	-.39***	-.58***	--			
12 Implicit Racial Bias	-.21***	-.23***	-.56***	.72***	--		
13 Cases	.08*	.46***	.47***	-.36***	-.29***	--	
14 Deaths	.21***	.29***	.32***	-.26***	-.11**	.53***	--
*Mean*	3.48	11.58	9.75	-.14	.29	1466.37	38.12
*SD*	4.34	13.21	11.60	.40	.07	941.35	42.20

**Notes**.

*p < .05.

**p < .01.

***p < .001.

### Hypothesis testing

Results related to *Cases* are presented in Table 2. The controls accounted for 53% of the variance, as shown in Model 1. The effects of *Explicit Racial Attitudes* are captured in Model 2, explaining a unique 2% of the variance beyond the effects of the controls. Consistent with Hypothesis 1, *Explicit Racial Attitudes* were positively related to *Cases*. Similarly, as seen in Model 4, *Implicit Racial Attitudes* held a significant, positive association with Cases, explaining 1% unique variance; thus, Hypothesis 3 was also supported.

**Table 2:** Effects of explicit racial attitudes and implicit racial attitudes on COVID-19 cases, January 22, 2020 to August 31, 2020.

	Model 1		Model 2		Model 3		Model 4		Model 5	
Variable	*B*	*SE*	*B*	*SE*	*B*	*SE*	*B*	*SE*	*B*	*SE*
Fair or Poor Health	351.44***	60.54	333.97***	59.18	305.99***	59.12	318.20***	60.11	307.00***	59.87
Uninsured	203.61***	41.50	200.11***	40.53	198.62***	40.18	204.86***	40.94	197.39***	40.77
Unemployed	-13.43	38.17	-12.33	37.27	-14.31	36.95	-20.56	37.67	-22.26	37.46
Food Insecure	-243.43***	52.32	-168.73**	52.42	-186.35***	52.18	-183.77**	53.06	-194.28***	52.86
Log Household Income	49.64	49.20	73.58	48.19	60.12	47.91	57.62	48.56	53.76	48.30
Percent 65 or Older	-42.68	35.13	-45.32	34.30	-46.28	34.01	-41.92	34.65	-36.73	34.49
Percent Female	-204.04***	53.70	-181.55**	52.56	-201.68***	52.37	-225.46***	53.15	-252.86***	53.54
Percent Asian	-3.11	18.05	16.38	17.89	7.07	17.90	6.92	17.92	-0.53	17.97
Percent Hispanic	271.40***	38.16	370.53***	40.40	363.56***	40.10	322.37***	39.09	324.20***	38.87
Percent Black	554.89***	41.30	679.27***	44.84	812.35***	56.28	644.71***	44.77	760.05***	57.26
Explicit Racial Attitudes			506.84***	79.83	432.47***	81.46				
Implicit Racial Attitudes							444.20***	91.79	398.50***	92.37
PB × Explicit					182.21***	47.25				
PB × Implicit									194.32**	60.67

**Notes**.

*p < .05.

**p < .01.

***p < .001. PB = Percent Black. Explicit = Explicit Racial Attitudes. Implicit = Implicit Racial Attitudes. Model 1 Δ*R*^2^ = .53, *p* < .001. Model 2 Δ*R*^2^ = .02, *p* < .001. Model 3 Δ*R*^2^ = .01, *p* < .001. Model 4 Δ*R*^2^ = .01, *p* < .001. Model 5 Δ*R*^2^ = .01, *p* = .001.

**Fig 1:**
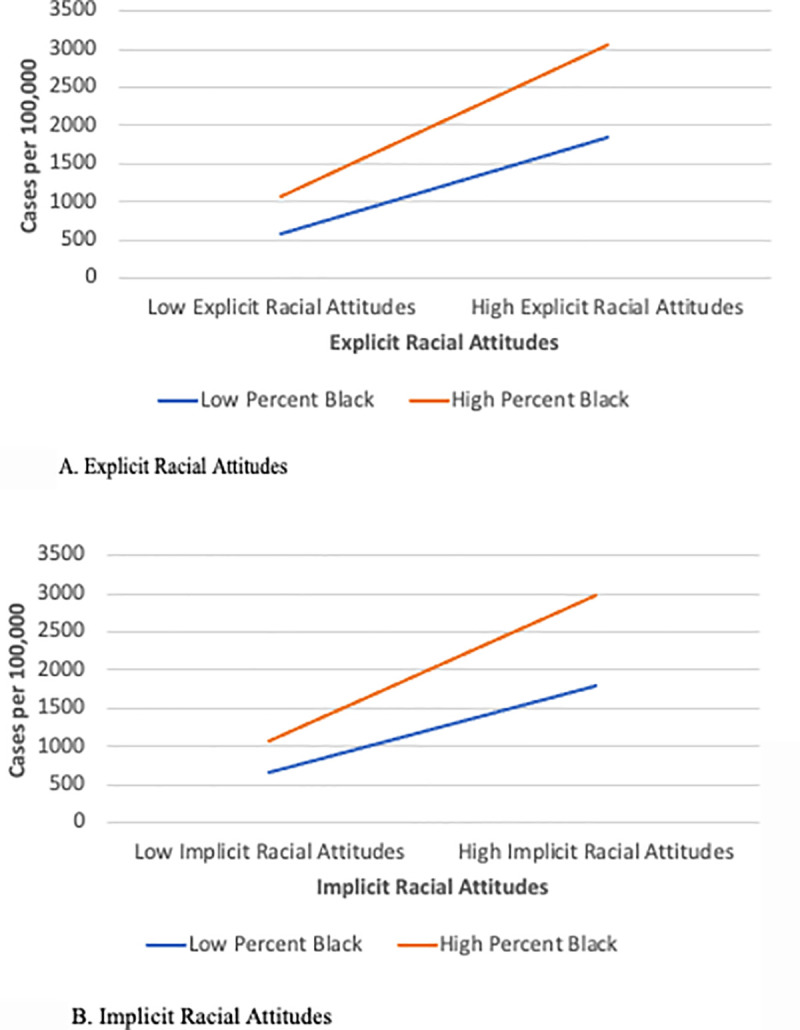
Relationships among percent black county residents, explicit racial attitudes, implicit racial attitudes, and COVID-19 cases per 100,000 residents for January 22, 2020 to August 31, 2020. A. Explicit racial attitudes. B. Implicit racial attitudes.

Table 1 also provides the tests for the moderating effects of explicit and implicit racial attitudes. As seen in Model 4, the *Percent Black* × *Explicit Racial Attitudes* interaction was significant. We then plotted the interaction (Fig 1A) and computed simple slopes, following Cohen et al.’s [47] guidelines. Results demonstrate that the relationship between *Percent Black* and *Cases* was stronger when *Explicit Racial Attitudes* were high (*B* = 994.56, *SE* = 93.06, *p* < .001) relative to when they were low (*B* = 630.14, *SE* = 42.25, *p* < .001). Thus, Hypothesis 5 was supported. We observed similar support for Hypothesis 6 (see Model 5 of Table 1). The relationship between *Percent Black* and *Cases* was stronger when *Implicit Racial Attitudes* were high (*B* = 994.56, *SE* = 93.06, *p* < .001) relative to when they were low (*B* = 630.14, *SE* = 42.25, *p* < .001). The relationship is illustrated in Fig 1B.

Table 3 provides the results for *Deaths*. The controls accounted for 33% of the variance. The main effects of Explicit Racial Attitudes were not significant (see Model 2), nor were the *Percent Black* × *Explicit Racial Attitudes* (see Model 4). Thus, Hypotheses 2 and 6 were rejected. On the other hand, Hypothesis 4 was supported, as *Implicit Racial Attitudes* held a positive, significant association with *Deaths* (see Model 3). Finally, the *Percent Black* × *Implicit Racial Attitudes* interaction term was not significant (see Model 5), so Hypothesis 8 was rejected.

**Table 3:** Effects of explicit racial attitudes and implicit racial attitudes on COVID-19 deaths, January 22, 2020 to August 31, 2020.

	Model 1		Model 2		Model 3		Model 4		Model 5	
Variable	*B*	*SE*	*B*	*SE*	*B*	*SE*	*B*	*SE*	*B*	*SE*
Fair or Poor Health	18.32***	3.50	18.02***	3.50	17.72***	3.52	16.68***	3.49	16.34***	3.49
Uninsured	-8.41***	2.40	-8.47***	2.39	-8.48***	2.40	-8.35***	2.37	-8.57***	2.38
Unemployed	5.90**	2.21	5.92**	2.20	5.90**	2.20	5.55*	2.19	5.50*	2.18
Food Insecure	-13.66***	3.02	-12.38***	3.10	-12.57***	3.11	-10.70**	3.08	-11.02***	3.08
Log Household Income	6.66*	2.84	7.07*	2.85	6.93*	2.86	7.06*	2.82	6.94*	2.81
Percent 65 or Older	6.03**	2.03	5.98**	2.03	5.97**	2.03	6.07**	2.01	6.22**	2.01
Percent Female	12.40***	3.10	12.78***	3.11	12.56***	3.12	11.34***	3.08	10.51**	3.12
Percent Asian	3.16**	1.04	3.49**	1.06	3.39**	1.07	3.65***	1.04	3.43**	1.05
Percent Hispanic	9.53***	2.21	11.23***	2.39	11.15***	2.39	12.06***	2.27	12.11***	2.27
Percent Black	19.59***	2.39	21.72***	2.65	23.19***	3.35	24.04***	2.60	27.51***	3.34
Explicit Racial Attitudes			8.69	4.72	7.87	4.86				
Implicit Racial Attitudes							22.02***	5.32	20.65***	5.38
PB × Explicit					2.01	2.82				
PB × Implicit									5.84	3.54

**Notes**.

*p < .05.

**p < .01.

***p < .001. PB = Percent Black. Explicit = Explicit Racial Attitudes. Implicit = Implicit Racial Attitudes. Model 1 Δ*R*^2^ = .33, *p* < .001. Model 2 Δ*R*^2^ = .00, *p* = .06. Model 3 Δ*R*^2^ = .00, *p* = .476. Model 4 Δ*R*^2^ = .01, *p* < .001. Model 5 Δ*R*^2^ = .01, *p* = .10.

We offer a summary of the findings in Table 4. Finally, we conducted a series of analyses to examine whether our decisions about the number of participants per county, the use of weights, or potential outliers impacted the pattern of results. As previously noted, we examined the hypotheses under several stipulations, as we: (a) limited the data aggregation of county-level data to those counties with at least 50 respondents; and (b) weighted the analyses using the log of the invariance variance weights on standard errors of the IAT and Thermometer scores. To determine whether these choices impacted the variance explained or interpretation of the results, we conducted additional analyses with different county participation cutoffs with the weights applied (*n* ≥ 100, and *n* ≥ 150), as well as analyses at the *n* ≥ 50 without the weights applied. Though there were slight variations, the overall pattern of results and variance explained was consistent across the cutoff points for participants per county. Further, unweighted regression analyses—that is, those not taking into account the differences in participants per county—resulted in less explanatory power. Table 1 also shows considerable variability in the number *Cases*. A Boxplot in SPSS showed four extreme outliers, or values more than three times the interquartile range. There were no such cases for *Deaths*. We computed a separate analysis for *Cases* excluding the extreme outliers, and the interpretation of the results remained the same.

**Table 4:** Summary of hypotheses and supplemental analyses.

Prediction	Finding
H1: Explicit racial attitudes will be positively related to COVID-19 cases.	Supported
H2: Explicit racial attitudes will be positively related to COVID-19 deaths.	Not Supported.
H3: Implicit racial attitudes will be positively related to COVID-19 cases.	Supported.
H4: Implicit racial attitudes will be positively related to COVID-19 deaths.	Not Supported.
H5: Explicit racial attitudes will interact with the percent of Black county residents to predict COVID-19 cases.	Supported.
H6: Explicit racial attitudes will interact with the percent of Black county residents to predict COVID-19 deaths.	Not Supported.
H7: Implicit racial attitudes will interact with the percent of Black county residents to predict COVID-19 cases.	Supported.
H8: Implicit racial attitudes will interact with the percent of Black county residents to predict COVID-19 deaths.	Not Supported.

## Discussion

The purpose of this research was to extend the existing scholarship related to race and COVID-19 by empirically considering the role of explicit and implicit racial attitudes. Results of the study showed that counties with high percentages of Black and Hispanic residents were also those that experienced more COVID-19 cases and deaths. These findings are consistent with previous researchers who have shown that a link between race and COVID-19 risk [1–4]. We observed as much from a bivariate perspective, and the relationships remained even after taking into account other control variables, including county demographics, subjective well-being, median household income, food security, percent uninsured, and percent unemployed. Results align with those from Lassale et al. [5], who observed racial differences in COVID-19-related hospitalizations even after taking into account personal and neighborhood factors.

These findings, though important, only tell part of the story, however. We also found that even after taking into account race and other county-level factors, aggregate measures of explicit racial attitudes and implicit racial bias provided additional explanatory evidence. The main effects of both racial attitudes variables were evident, as both were significant, positive predictors of COVID-19 cases. Importantly, however, these effects were qualified by a significant interaction with the percent of Black count residents. Specifically, the relationship between the percent of Black county residents and COVID-19 cases was stronger when explicit racial attitudes and implicit racial attitudes, respectively, were high relative to when they were low.

For COVID-19 deaths, however, the results were more equivocal. An increase in county-level implicit racial attitudes was linked with more COVID-19 deaths. However, the effects of explicit racial attitudes and the interaction of either racial attitudes term with the percent of Black county residents were not significant.

These collective findings point to the importance of county-level measures of racial attitudes and biases, especially when it comes to predicting COVID-19 deaths. Results buttress Payne et al.’s [19,20] arguments that aggregate measures of prejudice and bias can take on a shared property at the community level and effectively explain racial disparities. They also reinforce Blair and Brondolo’s [23] argument that community-level measures of racial attitudes and biases are likely to offer new insights into factors that predict health outcomes. Whereas others have identified direct effects [24–26], our results suggest county-level racial attitudes and biases can play important moderating roles. Put another way, a high percentage of Black residents is more likely to be associated with COVID-19 cases under some conditions (i.e., high levels of explicit racial attitudes and implicit racial attitudes) than others.

The next question is: why do these effects occur? Our controls accounted for a large portion of the variance in both COVID-19 cases and deaths. They also help rule out some potential explanations, including demographics, the health of the residents, the levels to which they are insured, have access to food, employed, and have financial resources. After taking into account these possibilities, it is possible that county-level racial attitudes are reflective of systemic forms of racism [49]. From this perspective, racial attitudes captured at the aggregate level represent the bias of crowds [20] and are emblematic of deeper biases that are embedded into systems within society. From this perspective, collective racial attitudes might give rise to racially biased societal arrangements, including those in the area of religion, business, education, criminal justice, and of particular importance to this study, healthcare. Payne et al. further argued that “In addition to functioning as a marker of systemic prejudice, implicit bias can also serve as a mechanism that translates systemic prejudice into individual discrimination” (p. 239). These dynamics would explain why, for example, implicit racial attitudes at the aggregate level are linked with racial differences in police shootings [27], punishments of Black school children [28], and various health-related outcomes [24–26]. The biases are learned and maintained over time such that the inequalities perpetuate. One apt illustration comes in the area of machine learning, where racial inequalities in healthcare over time have resulted in racially biased algorithms used to manage population health [50].

### Public health implications

There is still much to learn about the impact of COVID-19 on our nation’s health and wellbeing, which spans multiple dimensions and include more than just our physical health. COVID-19 illness and death continue to illuminate underlying structural inequalities that disproportionately affect racial and ethnic minorities. This pandemic further exposes interpersonal factors that may further exacerbate the disproportionate burden of chronic disease and premature death among US Black people. Our findings underscore the importance of raising awareness about the negative health consequences of explicit racial attitudes and implicit racial attitudes. We contribute to the evidence-base needed to support the need for and inform the development of training programs that will be aimed at increasing culture awareness and sensitivity among health care professionals.

### Limitations and future directions

Though our study makes several important contributions, we also note potential limitations. First, the direct effects of explicit racial attitudes and implicit racial attitudes explained 1–2% unique variance beyond the controls, and the interaction terms explained 1% unique variance. Some might consider the effects small. We acknowledge that possibility and also note (a) the direct and interactive effects of explicit racial attitudes and implicit racial bias were on top of a sizeable portion of variance explained by the controls (ranging from 33% to 53%), and (b) the percent variance explained by the moderators is consistent with social science researchers [51]. Second, we had complete data for 26% of the counties in the US, but this also means than we do not have information about the other 74%. The results could, therefore, vary if the additional cases were considered.

Finally, we note several opportunities for future research. First, the scholarship related to and understanding of COVID-19 continues to advance [34], but there is still much to learn. Our county-level approach proved to be efficacious, as we identified factors that contributed to more cases and deaths. The alternative approach is also needed, where researchers identify county- and community-level factors that help mitigate the number of COVID-19 cases and deaths. From a general public health standpoint, we echo Blair and Brondolo’s [23] sentiments, encouraging continued research related to the community-level effects of explicit racial attitudes and implicit racial biases.

## Supporting information

S1 File (SAV)
